# Gromwell (*Lithospermum erythrorhizon*) Attenuates High-Fat-Induced Skeletal Muscle Wasting by Increasing Protein Synthesis and Mitochondrial Biogenesis

**DOI:** 10.4014/jmb.2311.11034

**Published:** 2023-12-30

**Authors:** Ji-Sun Kim, Hyunjung Lee, Ahyoung Yoo, Hang Yeon Jeong, Chang Hwa Jung, Jiyun Ahn, Tae-Youl Ha

**Affiliations:** 1 Aging and Metabolism Research Group, Korea Food Research Institute, Wanju-gun, Jeollabuk-do 55365, Republic of Korea; 2 Department of Biotechnology, College of Life Science and Biotechnology, Korea University, Seoul 02841, Republic of Korea; 3 BK21 FOUR Institute of Precision Public Health, Interdisciplinary Program in Precision Public Health, Korea University, Seoul 02841, Republic of Korea; 4 Department of Food Biotechnology, University of Science and Technology, Wanju-gun, Jeollabuk-do 55365, Republic of Korea

**Keywords:** Gromwell (*Lithospermum erythrorhizon*), sarcopenic obesity, muscle atrophy, muscle mass, muscle performance, mitochondrial biogenesis

## Abstract

Gromwell (*Lithospermum erythrorhizon*, LE) can mitigate obesity-induced skeletal muscle atrophy in C2C12 myotubes and high-fat diet (HFD)-induced obese mice. The purpose of this study was to investigate the anti-skeletal muscle atrophy effects of LE and the underlying molecular mechanism. C2C12 myotubes were pretreated with LE or shikonin, and active component of LE, for 24 h and then treated with 500 μM palmitic acid (PA) for an additional 24 h. Additionally, mice were fed a HFD for 8 weeks to induced obesity, and then fed either the same diet or a version containing 0.25% LE for 10 weeks. LE attenuated PA-induced myotubes atrophy in differentiated C2C12 myotubes. The supplementation of LE to obese mice significantly increased skeletal muscle weight, lean body mass, muscle strength, and exercise performance compared with those in the HFD group. LE supplementation not only suppressed obesity-induced skeletal muscle lipid accumulation, but also downregulated TNF-α and atrophic genes. LE increased protein synthesis in the skeletal muscle via the mTOR pathway. We observed LE induced increase of mitochondrial biogenesis and upregulation of oxidative phosphorylation related genes in the skeletal muscles. Furthermore, LE increased the expression of peroxisome proliferator-activated receptor-gamma coactivator-1 alpha and the phosphorylation of adenosine monophosphate-activated protein kinase. Collectively, LE may be useful in ameliorating the detrimental effects of obesity-induced skeletal muscle atrophy through the increase of protein synthesis and mitochondrial biogenesis of skeletal muscle.

## Introduction

Sarcopenia is characterized by the age-related decrease in muscle mass and function. Obesity is a chronic and multifactorial disease characterized by the excessive accumulation of body fat [1]. These two diseases share common etiologies of pathogenesis, such as sedentary lifestyle, hormones, and immunological variables, which may operate synergistically to enhance the risk of adverse health consequences [2]. Sarcopenic obesity is defined as sarcopenia accompanied by an increase in the amount of adipose tissue and is prevalent in elderly people who are simultaneously sarcopenic and obese [3]. According to a longitudinal study, it was found that visceral obesity is associated with a reduction in skeletal muscle mass [4]. The global incidence of sarcopenic obesity among adult individuals is experiencing a notable escalation.

Skeletal muscles account for 40% of the body mass and aids in locomotion, postural support, glucose uptake, and fatty acid oxidation. They also play a vital role in regulating metabolism [5, 6]. Obesity is correlated with skeletal muscle loss, dysfunction, and muscle atrophy. Obese people with low muscle mass have an increased risk of death from various cancers, cardiovascular disease, and renal disease. As a result, the negative effects of decreased muscle mass are magnified in the context of obesity [7, 8]. The consumption of a diet that is rich in fats, particularly those containing high levels of saturated fatty acids like palmitic acid (PA), has been found to contribute to weight gain and is linked to the development of several metabolic disorders, such as insulin resistance and type II diabetes mellitus [9]. In a previous study, we showed that saturated fatty acids accumulate ectopically in skeletal muscle during obesogenic sarcopenia [10].

Protein homeostasis (proteostasis) is important to maintain skeletal muscle mass through the regulation of the balance between protein synthesis and degradation. Muscle atrophy occurs as a consequence of diminished protein synthesis and heightened protein degradation [11]. Muscle atrophy stressors such as inactivity, unloading, oxidative stress and inflammation induce the expression of E3 ubiquitin ligases, MuRF1 and Atrogin1 [12, 13]. The increased expression of MuRF1 and Atrogin1 is responsible for the shift of proteostasis to net degradation. The muscle atrophy is also associated with mitochondrial damage including loss of mitochondria, change of morphology, increase of oxidative stress, and impaired mitochondrial function [14]. Mitochondrial reactive oxygen species (ROS) triggers muscle atrophy signaling pathway for proteolytic activation [15].

*Lithospermum erythrorhizon* (LE), also known as gromwell, is a medicinal plant that grows in Asian countries and has an extensive history dating back to China’s Ming dynasty. LE has been utilized in traditional herbal medicine and as a dye for staining fabrics and as a food colorant [16]. LE possesses anti-tumor and anti-fungal properties and is externally applied to treat superficial wounds, including burns, frostbite, and hemorrhoids. The root of the LE contains significant amounts of shikonin derivatives, identifiable by the distinctive red color of the n naphthoquinone derivatives they produce [17]. Shikonin derivatives, identified as the active components of LE, exhibit various biological activities. In previous studies, we demonstrated that LE extract exhibits an anti-obesity effect in vivo [18] and shikonin has been identified as the functional compound for obesity and hepatic steatosis [19]. Additionally, various studies have reported that LE exerts antioxidant activity [20], antimicrobial activity [21], skin protection [22], and anticancer activity [23]. Despite these findings, studies on the improvement of muscle atrophy have not been conducted.

In this study, we investigated whether LE could improve obesity-associated muscle atrophy. We treated PA-induced myotubes and high-fat diet (HFD)-induced sarcopenic obese mice. We examined muscle performance using grip strength and a treadmill. Lean body mass and muscle mass were measured. We also analyzed markers related to muscle protein metabolism and mitochondrial content.

## Materials and Methods

### Sample Preparation

Dried LE was purchased from Youngju (Republic of Korea). LE extract was prepared as described previously [18]. Briefly, dried LE was soaked in 80% ethanol equivalent to 10 times the sample weight at room temperature for 12 h. This was repeated twice. The ethanol extracts were filtered through a filter paper (no. 2; pore size, 5 μm; Advantec MFS, Inc., Japan). After the ethanol and water evaporated, the solid contents of the final LE extract were 0.37 ± 0.05 g/ml.

### Cell Culture

Murine C2C12 myoblast was obtained from the American Type Cell Culture Collection (ATCC, USA). The C2C12 myoblasts were maintained in growth medium containing Dulbecco’s Modified Eagle’s medium (DMEM; HyClone, USA) supplemented with 10% fetal bovine serum (FBS; HyClone), 100 U/ml penicillin, and 100 μg/ml streptomycin (PS; Invitrogen, USA) in a humidified incubator kept at 37°C and 5% CO_2_. C2C12 myoblast cells begin to express myogenin upon almost reaching 100% confluency, and it upregulates after the switch to the differentiation medium for five days to matured myotubes [24]. Therefore, C2C12 differentiation to matured myofibers and PA induction occurred as previously described [25]. To induce myogenic differentiation, the C2C12 myoblasts were seeded at the density of 1 × 10^5^ cells/well in 6-well culture dishes. After reaching 95%confluence, the growth medium was replaced with a differentiation medium of DMEM supplemented with 2%horse serum (HS; HyClone) and 1% PS to induce myogenic differentiation. The differentiation medium was replaced with fresh medium daily. After 4 days, the myotubes were pretreated with LE or shikonin (Sigma Aldrich, USA) for 24 h, followed by treatment with 500 μM PA (Sigma Aldrich) for an additional 24 h. Bovine serum albumin (BSA; Sigma-Aldrich)-bound PA was prepared as described previously [26].

### Immunocytochemistry Analysis

Immunostaining for myosin heavy chain (MHC) was visualized in myotubes using immunofluorescence as previously described by Choi *et al*. [10]. Cells were plated on glass coverslips in 6-well dishes. After 24 h of PA treatment, the cells were washed with phosphate-buffed saline (PBS) and fixed in 4% paraformaldehyde in PBS. After permeabilization with 0.1% saponin, the slides were blocked with 1% BSA and incubated with an anti-MHC mouse monoclonal antibody (MF20; Developmental Studies Hybridoma Bank (DSHB), USA; 1:300 dilution) for overnight at 4°C. This was followed by incubation with Alexa-546-conjugated anti-mouse IgG (Molecular Probes, USA), and the nuclei were stained with 4’,6-diamidino-2-phenylindole dihydrochloride (DAPI; Molecular Probes). Images were captured using a fluorescence microscope (Olympus IX71, Japan). All cell nuclei and nuclei within myotubes were counted using the NIH Image J software (National Institute of Health, USA). The fusion index was measured as previous described [27].

### RNA Extraction and Quantitative Reverse-Transcription PCR

Cells and skeletal muscle tissues were excised, snap-frozen, and stored at -80°C before analysis. Total RNA was isolated using NucleoSpin RNA II (Macherey-Nagel, Germany) and cDNA was generated using the ReverTra Ace qPCR-RT Master Mix kit (Toyobo, Japan). qRT-PCR was conducted on a ViiA7 system (Applied Biosystems, USA) using SYBR Green real-time PCR master mix (Toyobo). Relative mRNA expression levels were calculated after normalizing the values to 18S mRNA. The primers used for the qPCR assays are shown in Table S1.

### Protein Extraction and Western Blot

Cells and skeletal muscle tissues were lysed in ice-cold protein lysis buffer (RIPA, Thermo Fisher Scientific, USA) containing protease and phosphatase inhibitors (Thermo Fisher Scientific) as previously described by Lee *et al*. [27]. Protein concentrations were determined using a BCA assay kit (Thermo Fisher Scientific) according to the manufacturer’s instructions. Protein samples were separated by SDS-polyacrylamide gel and transferred to PVDF membranes (Bio-Rad, USA). The membranes were blocked in 5% skimmed milk, and then incubated at 4°C overnight with the primary antibody. The primary antibodies were as follow: MHC (MHC-T; MF20; mouse monoclonal antibody, 223 kDa, 1:1,000 dilution, DSHB), MHC slow (MHC-I; BA-F8; mouse monoclonal antibody, 220 kDa, 1:1,000 dilution, DSHB), MHC type IIA (MHC-IIA; SC-71; mouse monoclonal antibody, 223 kDa, 1:1,000 dilution, DSHB), MHC type IIB (MHC-IIB; BF-F3; mouse monoclonal antibody, 223 kDa, 1:1,000 dilution, DSHB), β-actin (sc-47778, mouse monoclonal antibody, 43 kDa, dilution 1:1,000, Santa Cruz Biotechnology Inc., USA), GAPDH (PA1-9046; goat polyclonal antibody, 37 kDa, Thermo Fisher Scientific), phospho-Akt (p-AKT; no. 9271; rabbit polyclonal antibody, 60 kDa, 1:1,000 dilution, Cell Signaling Technology Inc., USA), Akt (no. 9272; rabbit polyclonal antibody, 60 kDa, 1:1,000 dilution, Cell Signaling Technology), p70 S6 Kinase (S6K; no. 2708, rabbit monoclonal antibody, 70 kDa, 1:1,000 dilution, Cell Signaling Technology), phospho-p70 S6 Kinase (p-S6K; no. 9205, rabbit polyclonal antibody, 70 kDa, 1:1,000 dilution, Cell Signaling Technology), phospho-4E-BP1 (p-4EBP1; no. 2855l rabbit monoclonal antibody, 15 kDa, 1:1,000 dilution, Cell Signaling Technology), 4E-BP1 (4EBP1; no. 9452; rabbit polyclonal antibody, 15 kDa, 1:1,000 dilution, Cell Signaling Technology), MuRF1 (ab183094; rabbit polyclonal antibody, 40 kDa, 1:1,000 dilution, Abcam Inc., USA), Muscle atrophy F-box (*MAFbx*; sc-33782; rabbit polyclonal antibody, 42 kDa, 1:1,000 dilution, Santa Cruz Biotechnology), PGC-1α (ab106814; goat polyclonal antibody, 91 kDa, 1:1,000 dilution, Abcam), phospho-AMP-activated protein kinase (p-AMPK; no. 2535; rabbit monoclonal antibody, 62 kDa, 1:1,000 dilution, Cell Signaling Technology), and AMPK (no. 2793; mouse monoclonal antibody, 62 kDa, 1:1,000 dilution, Cell Signaling Technology). After washing three times with Tris-buffered saline with 0.1% Tween 20 (TBST), the membranes were incubated with either anti-mouse, anti-rabbit, or anti-goat IgG and HRP-linked secondary antibodies (1:5,000 dilution, Santa Cruz Biotechnology). Immunoreactive proteins were visualized by chemiluminescence reagent treatment and observed with G:BOX Chemi XX6 (Syngene Ltd., USA). All figures showing the results of the quantitative analysis performed using ImageJ include data from at least three independent experiments.

### Animals

Male C57BL/6N mice aged 4 weeks were purchased from ORIENT, Inc. (Republic of Korea). The mice were group-housed in plastic cages, with two or three mice per cage. The cages were placed in a room with controlled temperature (23 ± 1°C) and a 12-h light/dark cycle. The mice were given food and distilled water *ad libitum*. After 1 week of acclimation, the mice were fed a normal chow diet (8640 Teklad 22/5 Rodent Diet; Halan Laboratories, USA) or HFD (06414l, 60% calories as fat; Halan) for 8 weeks to induce diet-induced obesity. Mice fed a HFD were randomly divided into two groups (12 mice per group) and fed HFD or HFD + LE (0.25% w/w) for the additional 10 weeks. The compositions of the experimental diets were shown in Table S2. Body weight was recorded weekly and diet intake was recorded every 2 to 3 days. All animal experiments were approved by the Institutional Animal Care and Use Committee of the Korea Food Research Institute (KFRI-M-18001).

### Body Composition Analysis

Body composition was assessed in all mice using dual-energy X-ray absorptiometry (DXA) (InAlyzer; Medikors Co., Republic of Korea). The mice were placed on the scanner bed in the prone position, with the limbs and tail stretched away from the body and scanned according to the instructions for operating the InAlyzer system. After the scan, the body composition was calculated using the InAlyzer software.

### Measurement of Muscle Performance

For the grip strength test of the front paws, the mice from each group were evaluated three times using a grip strength meter (Bioseb, France) with a 3 min rest period between trials to prevent fatigue. To perform the endurance running test, mice were placed on a motorized, speed-controlled rodent treadmill system (Ugo Basile, Italy). After 2 days of acclimation, the mice ran on the treadmill at an inclination of 15° and underwent the following running program: 10 m/min for 20 min after which the speed was increased every 2 min by up to 2 m/min until exhaustion.

### Histological Analysis

Isolated gastrocnemius (GAS) skeletal muscle tissues were fixed in a buffer solution of 4% formalin and embedded in paraffin. Sections with a thickness of 4-μm were prepared and stained with hematoxylin and eosin (H&E). The stained areas were viewed using a light microscope at a magnification of ×200. Cross-sectional areas (CSA) were measured using ImageJ.

### Biochemical Analysis

To measure the skeletal muscle lipid content, total lipids were extracted with chloroform and methanol in GAS skeletal muscle tissue as described by Folch *et al*. [28]. To determine the skeletal muscle triacylglycerol (TG) content, dried lipid residues were dissolved in 1 ml of ethanol. For emulsification, Triton X-100 and sodium cholate solutions in distilled water were added to 200 μl of the dissolved lipid solution. TG content was immediately quantified using a commercial kit (Shinyang). Tumor necrosis factor-α (TNF-α) in serum was measured using a mouse ELISA kit (R&D Systems, USA).

### Measurement of Mitochondrial DNA (mtDNA) Content

The quantification of nuclear DNA and mtDNA content was conducted via qPCR. Genomic DNA was extracted from GAS skeletal muscle tissue using the DNeasy kit (Qiagen, USA). The mtDNA to nuclear DNA ratio served as an indicator of cellular mitochondrial content. To determine this ratio, Ct values were obtained for the *Cox5a* gene, which is encoded by mtDNA, and the 18s rRNA gene, which is encoded by nuclear DNA.

The relative mtDNA copy number was calculated by normalizing the copy number of the 18S rRNA gene. The *Cox5a* and 18S rRNA primers used for qPCR are shown in Table S1.

### Statistical Analysis

The results are expressed as the mean ± standard deviation (SD) for cell studies and the mean ± standard error of the mean (SEM) for animal studies. Statistical analyses were performed using the GraphPad Prism 8 software (USA). One-way analysis of variance (ANOVA) with Tukey’s *post-hoc* tests was used to compare quantitative data among the groups. *P* values less than 0.05 were considered statistically significant.

## Results

### Shikonin Attenuates PA-Induced Muscle Atrophy in C2C12 Myotubes

**Fig. 1:**
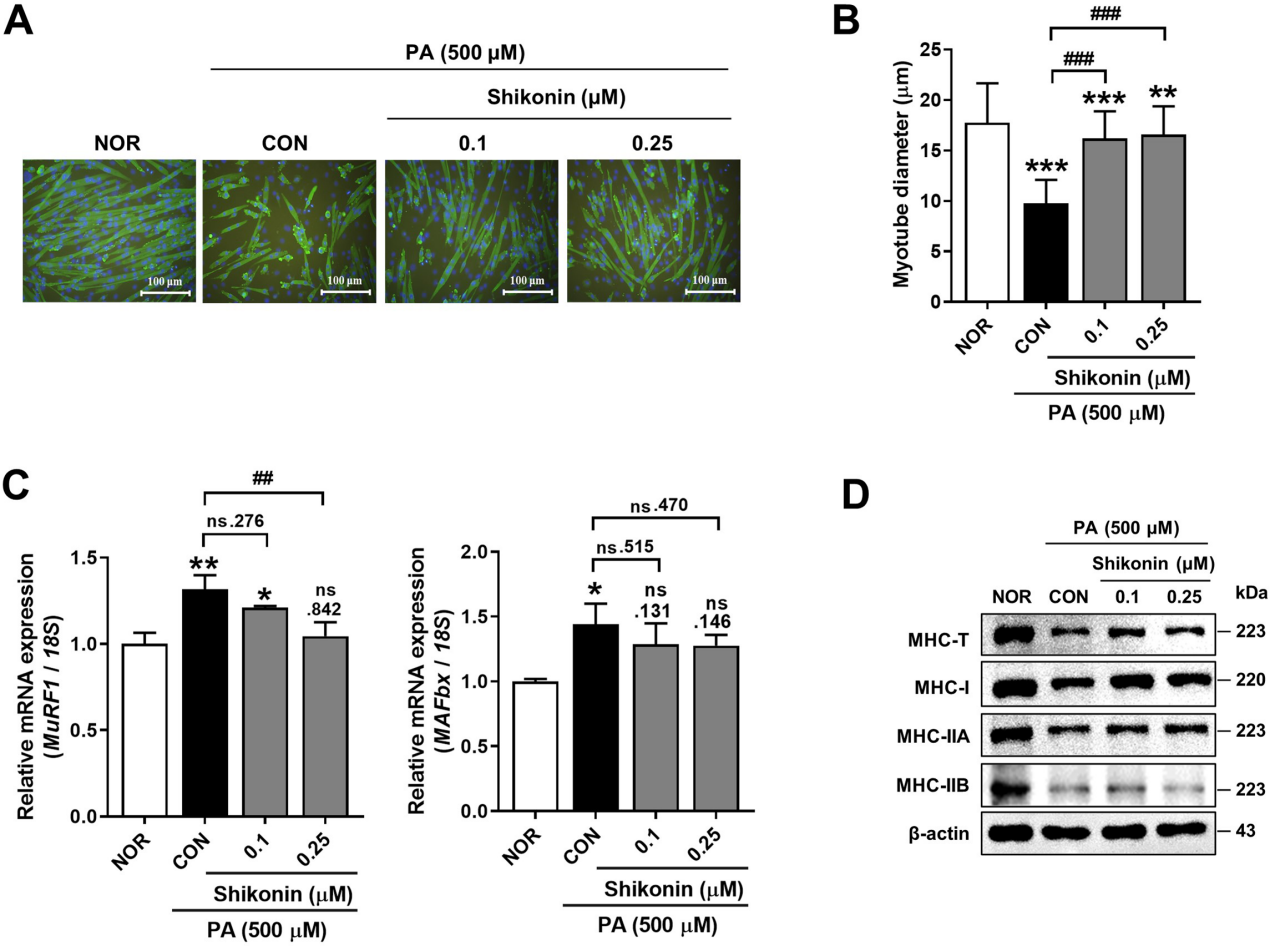
Shikonin attenuated palmitic acid (PA)-induced muscle atrophy in C2C12 myotubes. (**A**) Immunofluorescence staining for myosin heavy chain (MHC) 24 h after treatment. Scale bars represent 100 μm. (**B**) Quantification represents the diameter of myotube. (**C**) RT-qPCR analysis of the mRNA levels of *MAFbx* and *Murf1* in shikonin-treated C2C12 myotubes. (**D**) Immunoblotting for MHC-T, MHC-I, MHC-IIa, MHC-IIb, and β-actin of shikonin-treated C2C12 myotubes. All quantifications were performed in three independent experiments (*n* = 3). Error bars represent the standard deviation (SD). *p < 0.05, **p < 0.01 and ***p < 0.001 versus control (CON; PA treatment). Statistically significant differences were determined using one-way ANOVA followed by Tuckey’s *post-hoc* test.

Previously, we reported the anti-obesity effect of LE and found shikonin is the functional compound of LE [18]. In this study, we tested whether shikonin could attenuate the PA-induced C2C12 myotube atrophy. As shown in Fig. 1A and 1B, the average diameter of the normal group, which was 17.76 μm, decreased to 9.77 μm when PA alone was treated, but was restored to 16.20 and 16.60 μm by 0.1 and 0.25 μM shikonin treatments, respectively. It was observed that mRNA expression of *Murf1* and *MAFbx*, two muscle-specific E3 ubiquitin ligases, was increased after PA treatment on C2C12 myotubes. Shikonin treatment showed a tendency to reduce the expression of *MAFbx*, but there was no significant difference. However, mRNA expression of *Murf1* was significantly inhibited by 0.25 μM shikonin treatment (Fig. 1C). The protein expression levels of total and MHC isoforms were analyzed by western blot analysis. Exposure to PA significantly decreased the expression of total and MHC isoforms, which were increased by treatment of shikonin (Fig. 1D). From the above results, it was suggested that shikonin has the potential to prevent PA-evoked C2C12 myotube atrophy.

### LE Prevents PA-Induced Muscle Atrophy in C2C12 Myotube

**Fig. 2:**
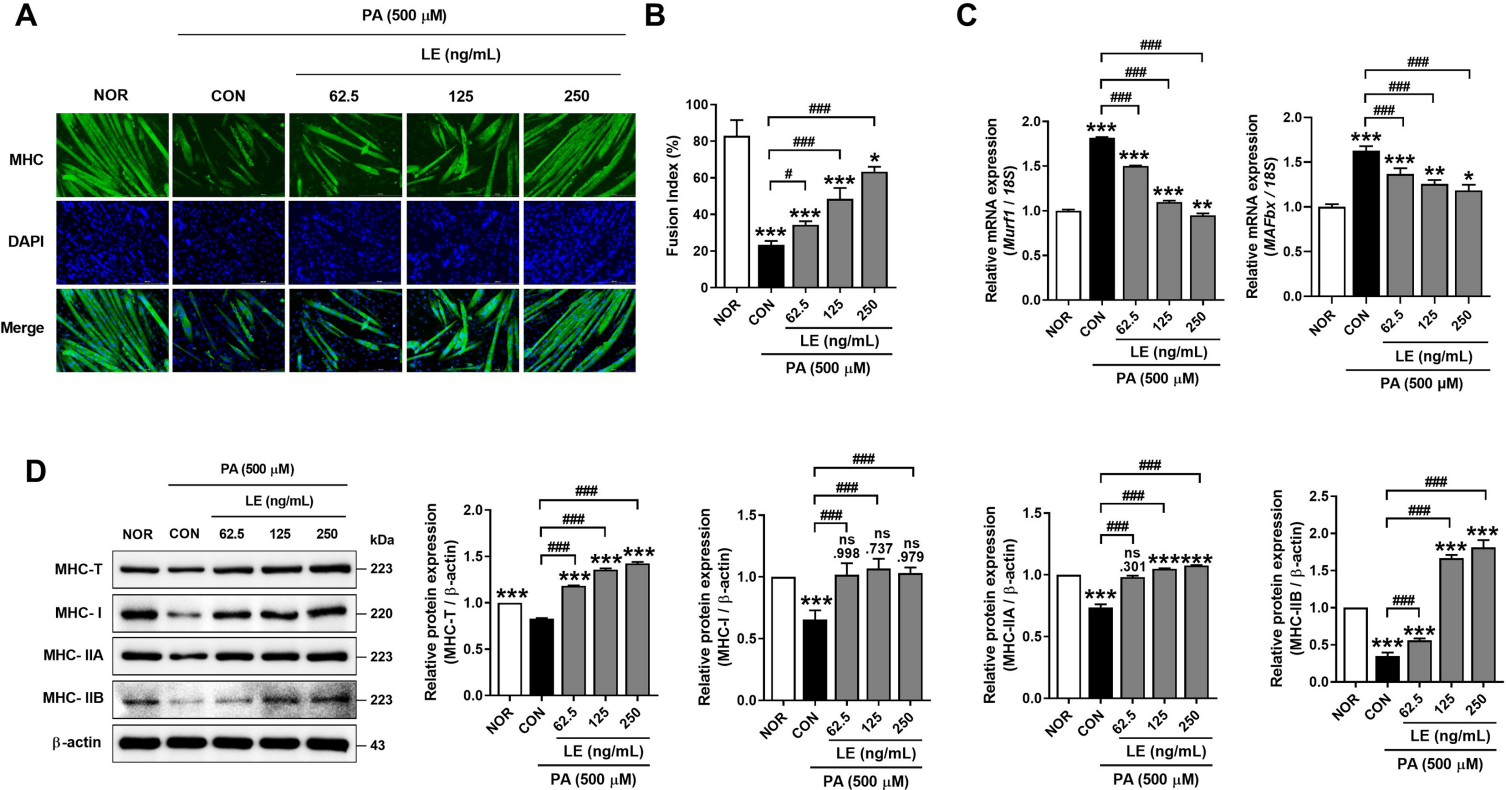
*Lithospermum erythrorhizon* (LE) prevents PA-induced muscle atrophy in C2C12 myotubes. (**A**) Immunofluorescence staining for MHC 24 h after treatment. Scale bars represent 50 μm. (**B**) Quantification represents the average percent of fusion index. (**C**) RT-qPCR analysis of the mRNA levels of *MAFbx* and *Murf1* in LE-treated C2C12 myotubes. (**D**) Immunoblotting for MHC-T, MHC-I, MHC-IIa, MHC-IIb, and β-actin of LE-treated C2C12 myotubes. Quantification represents the relation to NOR. All quantifications were performed in three independent experiments (*n* = 3). Error bars represent the standard deviation (SD). **p < 0.01 and ***p < 0.001 versus control (CON; PA treatment). Statistically significant differences were determined using one-way ANOVA followed by Tuckey’s *post-hoc* test.

Since we observed the protective effect of shikonin on myotube atrophy, we tested the effect of LE on PA-induced muscle atrophy in C2C12 myotubes. As shown in Fig. 2A, exposure to 500 μM PA decreased myotube diameter compared with normal cells, and treatment with LE inhibited PA-induced atrophy of C2C12 myotubes. The fusion index was measured as the percentage of the number of DAPI-stained nuclei located within MHC-positive myotubes to the total number of nuclei (Fig. 2B). The myotubes treated with PA showed a reduced fusion index compared with that of the normal group. However, LE treatment significantly increased the fusion index in a dose-dependent manner (Fig. 2B). Treatment with PA upregulated muscle atrophy markers such as *Murf1* and *MAFbx*, whereas treatment with LE effectively decreased these genes (Fig. 2C). Next, we measured the protein expression of total and MHC isoforms by western blot analysis (Fig. 2D). Exposure to PA significantly decreased the expression of total and MHC isoforms, which were increased after LE treatment (*p* < 0.001 vs. CON). These data suggested that LE prevented PA-induced myotubes atrophy.

### LE Ameliorates HFD-Induced Skeletal Muscle Atrophy in Mice

**Fig. 3:**
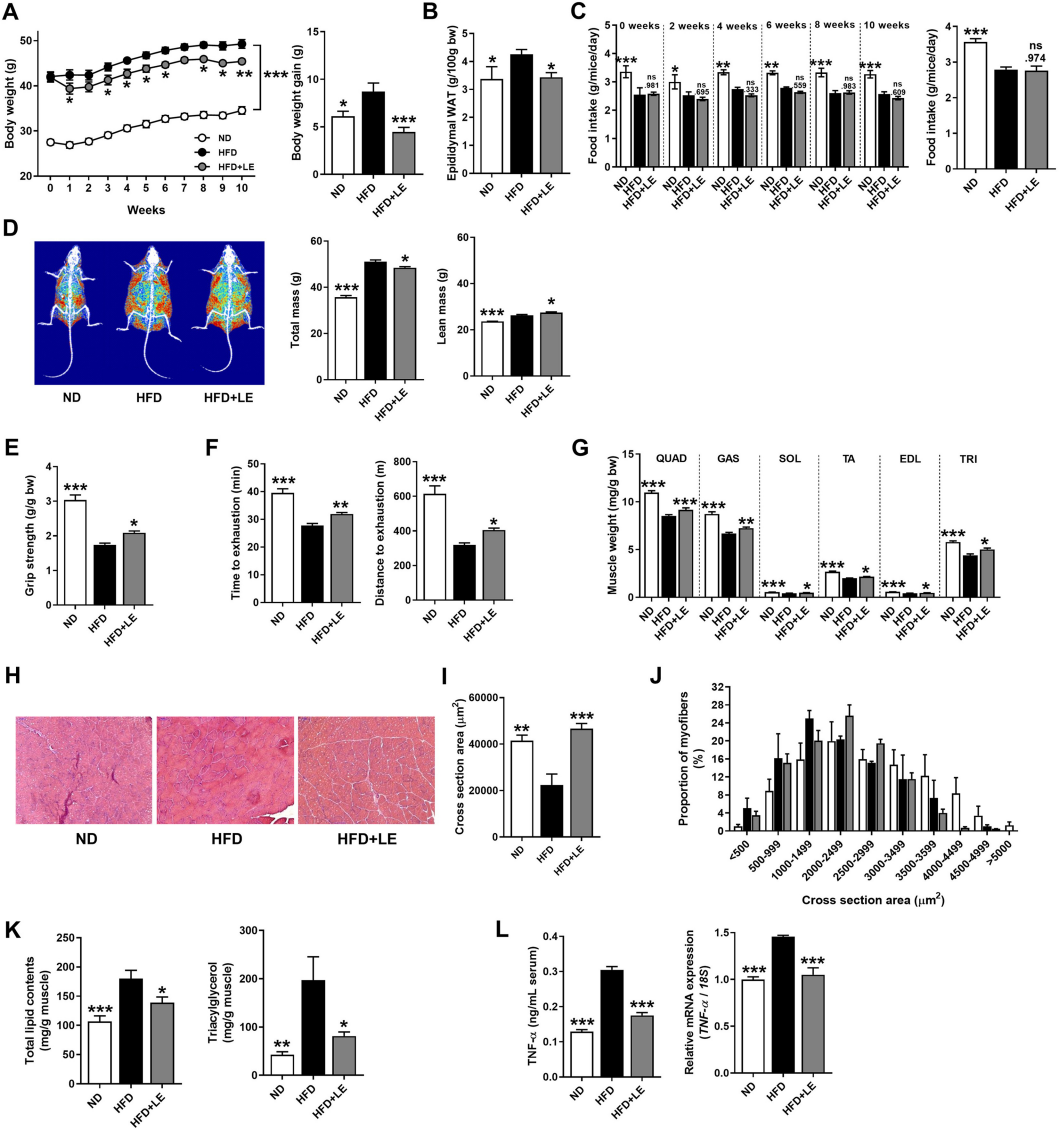
LE ameliorates skeletal muscle wasting induced by high-fat diet (HFD) in C57BL/6N mice. (**A**) Effects of LE on body weight (left panel) and body weight gain (right panel) for 10 weeks. (**B**) Epididymal white adipose tissue (WAT) weight. (**C**) Effects of LE on food intake (left panel) and average food intake (right panel) in a period of 10 weeks. (**D**) Representative dual-energy X-ray absorptiometry (DXA) images (left panel) and calculated total mass and lean mass (right panel). (**E**) Effects of LE on muscle strength. (**F**) Effect of LE on exercise endurance capacity. Distance (left panel) and time (right panel) to exhaustion of treadmill tests. (**G**) Measurement of isolated muscle weights. QUAD, quadriceps, GAS, gastrocnemius; SOL, soleus, TA, tibialis anterior; EDL, extensor digitorum longus; TRI, triceps brachii. (**H**) Representative hematoxylin and eosin (H&E) staining of muscle cross section. (**I**) Mean cross-sectional area of the GAS. (**J**) Frequency histograms and frequency of fibers for myofiber distribution. (**K**) The measurements of total lipid content (left panel) and triacylglycerol level (right panel) in the gastrocnemius muscle tissues. (**L**) The circulating level (left panel) and mRNA expression (right panel) of TNF-α in muscle tissue. Epididymal WAT and isolated muscle weights are represented in relation to whole body weight (g/100 g bw). Data are presented as mean ± standard error of the mean (SEM). *p < 0.05, **p < 0.01, and ***p < 0.001 versus HFD group. ND, normal diet fed group; HFD, high-fat diet fed group; HFD+LE, high-fat diet supplemented with 0.25% LE extract. Statistically significant differences were determined using one-way ANOVA followed by Tuckey’s *post-hoc* test.

Previously, we confirmed that LE supplementation has an anti-obesity effect in vivo [18]. To investigate the therapeutic effect of LE on obesity-associated reductions in muscle mass and function, mice were fed an HFD for 8 weeks to induced obesity. Subsequently, obese mice were fed an HFD containing LE (HFD+LE) for an additional 10 weeks. Supplementation with LE significantly reduced body weight gain during the experimental period (Fig. 3A). Additionally, the weight of epididymal white adipose tissue (WAT), representing abdominal fat, was significantly lower in the LE supplementation group compared to the HFD group (Fig. 3B). Notably, within the HFD groups, no significant changes in food intake were observed on LE supplementation during the experimental period (Fig. 3C). Previous study has demonstrated that LE supplementation has an anti-obesity effect, accompanied by a reduction in WAT when administered concurrently with a HFD [18]. Remarkably, the results of this study confirm that LE supplementation exhibits anti-obesity effect even in the context of diet-induced obesity. The body composition analysis showed LE treatment significantly decreased the total mass compared to that in the HFD group (Fig. 3D). However, lean mass significantly increased in the LE treatment group compared to that in the HFD group.

Next, we examined the effects of LE on muscle performance. The LE-supplemented group exhibited significant increases in muscle strength (Fig. 3E) and exercise performance (Fig. 3F) compared to the values reported for the HFD group. In addition, isolated skeletal muscle weights were significantly increased by LE supplementation (Fig. 3G). These data confirmed that LE inhibited HFD-induced decreases in muscle mass and impaired muscle function.

To investigate the effect of LE on HFD-induced muscle atrophy, we measured the CSA of the GAS skeletal muscle and found that supplementation with LE significantly increased CSA compared to the HFD group (Fig. 3H and 3I) (*p* < 0.001). In addition to the increase in GAS skeletal muscle fiber size, the proportion of larger myofibers was higher in the HFD+LE group than that in the HFD group (Fig. 3J). Intramuscular adiposity results in declines in muscle strength and quality [29]. We measured intramuscular fat content and observed that HFD-induced lipid accumulation in the GAS skeletal muscle was ameliorated in the LE-supplemented group (Fig. 3K). In addition, obesity is responsible for causing systemic low-grade inflammation, particularly by visceral fat, which excretes several different pro-inflammatory cytokines [30]. We observed that LE supplementation significantly decreased TNF-α levels in the GAS skeletal muscle and serum compared to the values reported for the HFD group (Fig. 3L). Taken together, our results indicated that LE alleviated HFD-induced skeletal muscle atrophy, reduced exercise capacity, and reduced lipid accumulation.

### LE Improves the Proteostasis in the Skeletal Muscle of HFD-Fed Mice

**Fig. 4:**
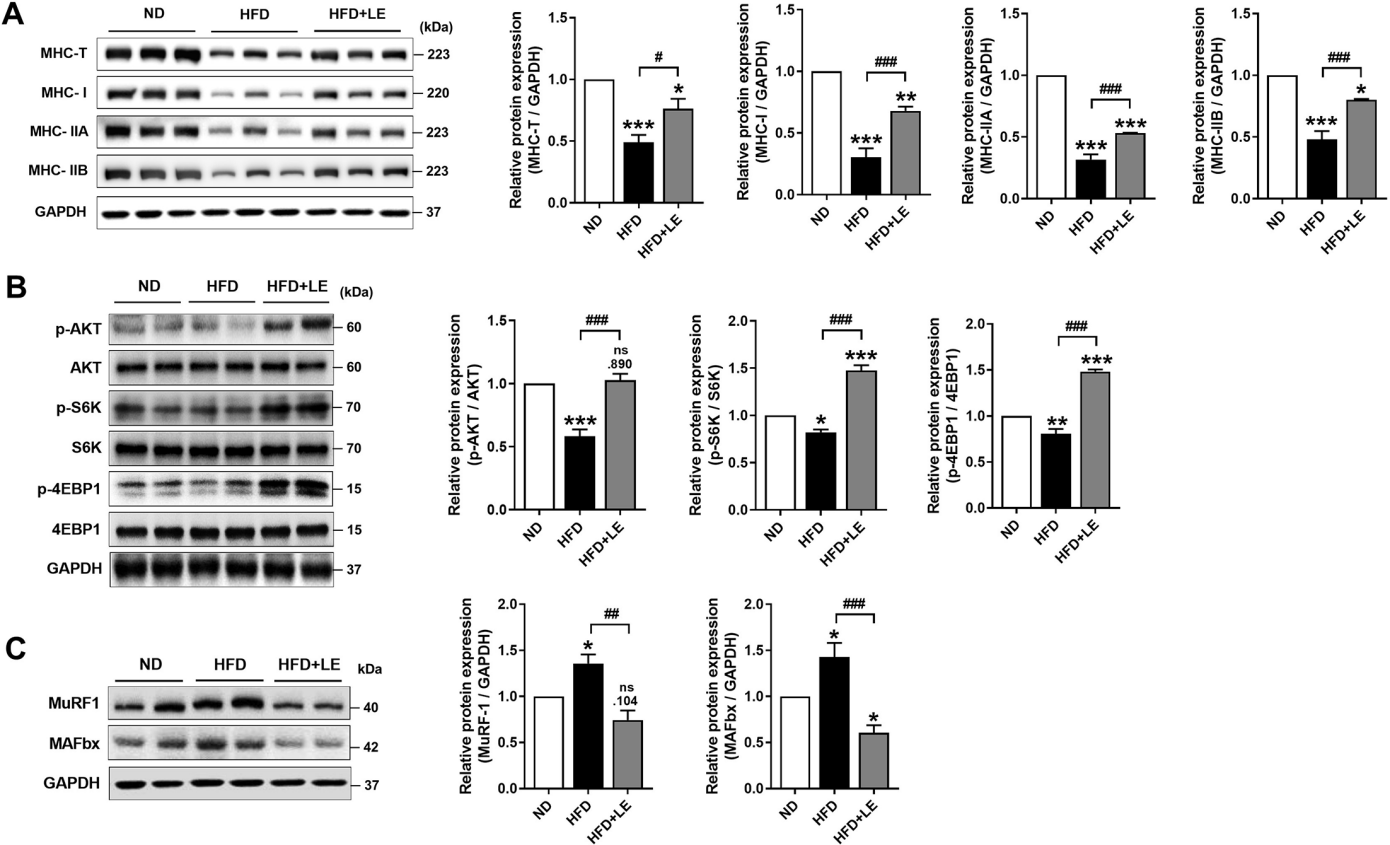
LE activates protein synthesis pathway and decreases atrophy markers in obese sarcopenic mice. (**A**) Western blots for expression of MHC isoforms in skeletal muscle (left panel) and the densitometric analysis (right panel). (**B**) Western blots for expression of AKT-mTOR pathway in skeletal muscle (left panel) and the densitometric analysis (right panel). (**C**) Western blots for expression of atrophy markers (left panel) and their densitometric analysis (right panel). Data are presented as mean ± standard error of the mean (SEM). *p < 0.05, **p < 0.01, and ***p < 0.001 versus HFD group. ND, normal diet fed group; HFD, high-fat diet fed group; HFD+LE, high-fat diet supplemented with 0.25% LE extract. Statistically significant differences were determined using one-way ANOVA followed by Tuckey’s *post-hoc* test.

We measured the protein expression of MHC, which is an important structural protein of muscle fibers, and observed decreased expression of total, type I, type IIA, and type IIB MHC isoforms in the GAS muscle tissues of the HFD group (Fig. 4A). However, LE supplementation significantly restored the HFD-induced decrease in MHC isoform levels.

To determine whether LE affects protein synthesis, we examined the Akt/mTOR signaling pathway, a critical pathway that regulates protein synthesis and muscle hypertrophy [31]. Western blot analysis demonstrated that supplementation with LE increased the phosphorylation of AKT and downstream signaling molecules of mTOR, such as S6K and 4EBP1 (Fig. 4B). Furthermore, LE supplementation markedly inhibited the HFD-induced increase in MuRF1 and *MAFbx* expression (Fig. 4C). These data indicate that LE improves proteostasis by increasing protein synthesis and decreasing protein degradation during obesity-associated muscle atrophy.

### LE Improves Mitochondrial Biogenesis in HFD-Fed Obese Mice

**Fig. 5:**
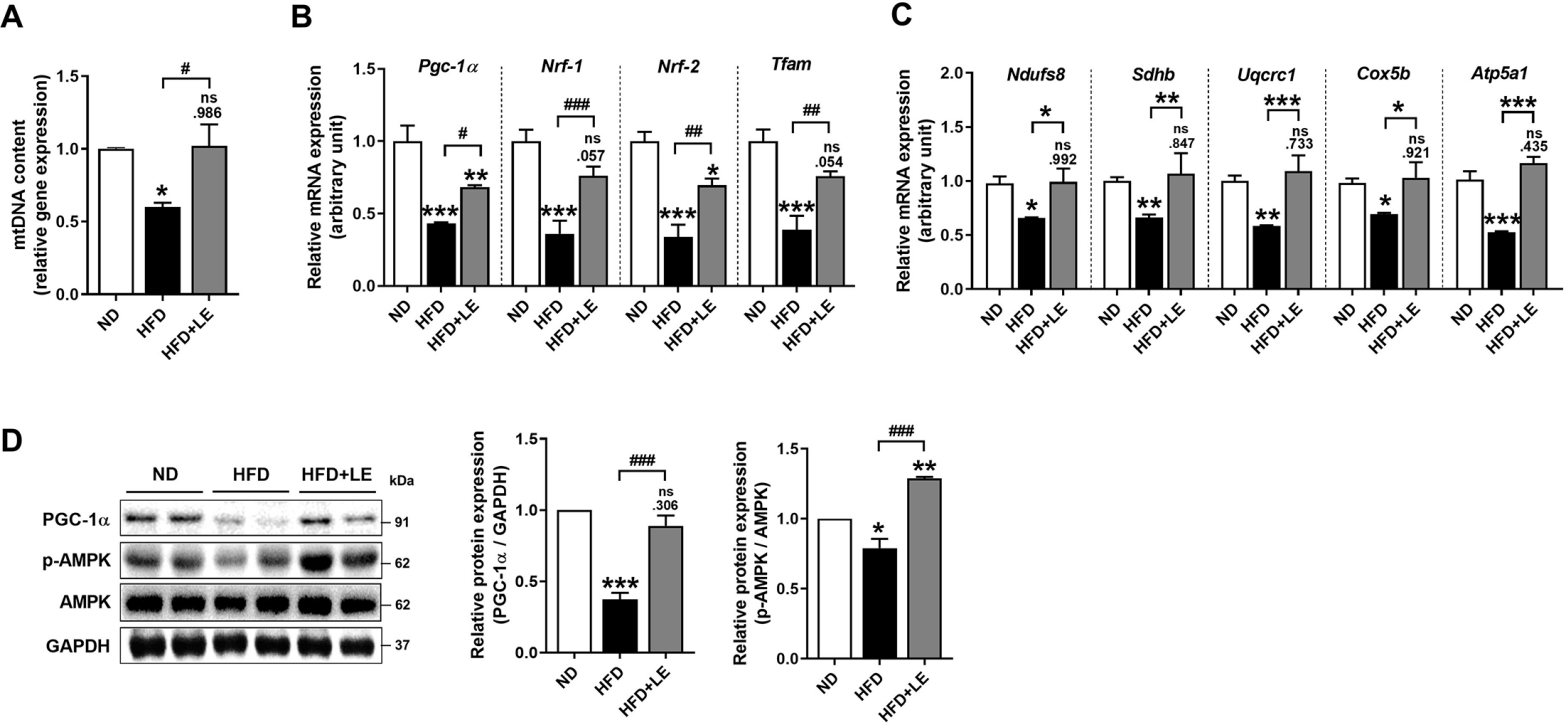
LE increased mitochondrial biogenesis through PGC-1α in obese sarcopenic mice. (**A**) Mitochondrial DNA (mtDNA) content in skeletal muscle of obese sarcopenic mice. (**B**) qRT-PCR analysis for the measurement of mRNA expression of mitochondria biogenesis regulated genes. (**C**) mRNA expressions of mitochondrial oxidative phosphorylation associated genes. (**D**) Western blot analysis for PGC-1α and AMPK phosphorylation (left panel) and their quantification (right panel) in skeletal muscle. Data are presented as mean ± standard error of the mean (SEM). *p < 0.05, **p < 0.01, and ***p < 0.001 versus HFD group. ND, normal diet fed group; HFD, high-fat diet fed group; HFD+LE, high-fat diet supplemented with 0.25% LE extract. Statistically significant differences were determined using one-way ANOVA followed by Tuckey’s *post-hoc* test.

We examined whether the LE-associated alleviation of HFD-induced muscle atrophy was related to the improvement of mitochondrial dysfunction. The mtDNA content was significantly increased in the skeletal muscle of the LE-treated group (Fig. 5A). We then measured the mRNA expression of mitochondrial biogenesis. LE supplementation significantly upregulated the mRNA levels of mitochondrial biogenesis-related genes, such as *Pgc-1α*, *Nrf-1* and *-2*, and *Tfam* (Fig. 5B). LE treatment also significantly increased the levels of OXPHOS genes such as NADH dehydrogenase [ubiquinone] iron-sulfur protein 8 (*Ndusf8* or Complex I), succinate dehydrogenase [ubiquinone] iron-sulfur subunit (*Sdhb* or Complex II), ubiquinol-cytochrome c reductase core protein 1 (*Uqcrc1* or Complex III), cytochrome c oxidase subunit 5b (*Cox5b* or Complex IV), and ATP synthase F1 subunit alpha (*Atp5a1* or Complex V) (Fig. 5C). Interestingly, we also found that PGC-1α was significantly elevated in GAS skeletal muscle upon LE supplementation. In addition, phosphorylation of AMPK was significantly increased in the LE-supplemented group compared to that reported for the HFD-fed group (Fig. 5D). These findings demonstrated that LE improves skeletal muscle mitochondrial activity and ameliorates HFD-induced muscle atrophy.

## Discussion

**Fig. 6:**
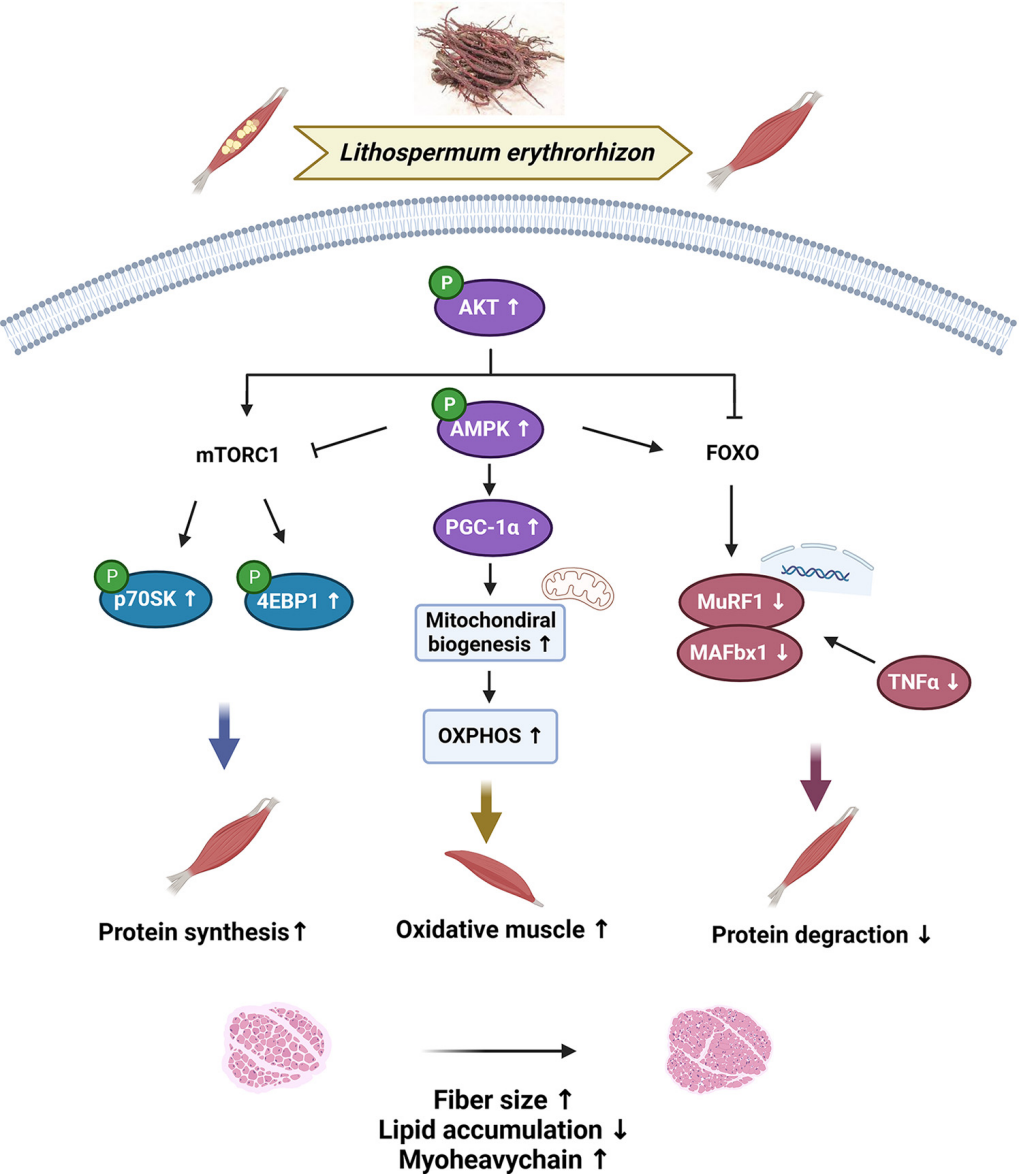
Schematic of the beneficial effects of LE on obesity induced skeletal muscle atrophy.

Sarcopenic obesity, which is characterized by increased amounts of adipose tissue and decreased muscle mass and strength, is more detrimental to frailty and mortality in the elderly than sarcopenia or obesity alone [32]. Thus, it is important to identify potential candidates that may be used to prevent obesity-associated sarcopenia and increase the quality of life. LE has long been used in traditional Asian medicine for the treatment of various diseases [33, 34]. Shikonin, the active component in LE, is a lipid-soluble naphtoquinone compound. Several studies have shown that it exhibits anti-cancer activity by cancer cell apoptosis in vitro and in vivo. Previously, we confirmed that a HFD supplemented with 0.25% or 0.5% LE significantly reduced the body weight gain, adipocyte enlargement, adipose tissue weight gain, serum and hepatic lipid, and adipogenesis gene expression in mice [18]. In addition, we confirmed that shikonin, the active compound of LE, reduced adipogenesis by reducing the expression of adipogenic genes [35], as well as reduced haptic lipid accumulation through activation of AMPK [19]. Furthermore, shikonin supplementation increased the fatty acid oxidation in liver and skeletal muscle of mice [36]. However, it remains unclear whether LE protects obesity-related skeletal muscle atrophy. In the present study, we showed that LE inhibits obesity-associated muscle atrophy by downregulating inflammatory cytokines and improving mitochondrial function (Fig. 6).

Using C2C12 myotubes that had been treated with PA to cause fat invasion, we created an environment similar to that of an obese condition. Free fatty acids evoke insulin resistance but also atrophy in skeletal muscles [37]. In particular, PA is enriched in HFD and is implicated in the development of sarcopenic obesity [38]. Exposure to saturated PA and non-unsaturated oleic acid induces lipotoxicity-induced myofiber loss in C2C12 myotubes [39]. Two E3 ubiquitin ligases, namely MAFbx/Atrogin1 and MuRF1, are part of the ubiquitin proteasome pathway utilized for protein degradation in muscle atrophy; therefore, these markers are considered the master genes of muscle atrophy [12]. MuRF1 degrades muscle structural proteins such as MHC, myosin light chain, and actin, whereas MAFbx is involved in growth- or survival-related pathways [40]. We found that LE and its active compound shikonin downregulated genes such as *Murf1* and *MAFbx* in PA-treated myotubes, leading to an increase in MHC expression.

A HFD is an important risk factor for sarcopenia and may be used to induce sarcopenia in animal models. HFD-fed mice showed fat accumulation in the muscles and loss of muscle mass [41] as well as damaged muscle regeneration ability and myogenic differentiation [42]. In this study, muscle atrophy was induced by feeding obese mice a HFD for 18 weeks and was confirmed by the loss of muscle mass, decrease in physical activity, and increase in body and tissue weight, including fat fads.

Despite the reduction in lean body mass, weight loss appears to be an effective intervention for the treatment of sarcopenic obesity. In a previous study, LE was shown to have anti-obesity activity by suppressing the expression of lipogenic genes in the liver and transcription factors in white adipose tissue [18]. Shikonin, a major component of LE, significantly improved lipid metabolism and lipogenesis in 3T3-L1 cells in vitro and HFD-induced obesity in vivo [18]. In the present study, we found that LE, as an anti-obesity candidate, improved sarcopenia with increased muscle function, mass, and activity. We therefore suggest LE as a novel candidate for the prevention of muscle atrophy.

Sarcopenia accompanied by obesity leads to further deficits in muscle mass and CSA [43]. We confirmed the decreased CSA of myofibers by HFD and found that LE increased the proportion of large fibers. Ectopic fat accumulation in skeletal muscles occurs in the form of intramyocellular lipids, which include lipid droplets within muscle cells and intermuscular adipose tissue distributed in the muscle interstitium or surrounding muscle fascicles [44]. The intramyocellular lipids stimulate inflammation and lipotoxicity [45]. Among various inflammatory cytokines, TNF-α is known to promote muscle wasting by increasing protein degradation and inhibiting muscle regeneration [46]. It was suggested that the decreased release and production of the inflammatory cytokine TNF-α resulted from reduced lipid accumulation in skeletal muscles by LE.

Muscle atrophy results in impaired proteostasis, increased protein degradation, and decreased protein synthesis. AKT regulates protein synthesis and degradation via the mTOR and FoxO families, respectively. The upregulation of *MAFbx* and MuRF1 is blocked by AKT phosphorylation via the inhibition of nuclear translocation of FoxO3 [47]. We found that LE induced an AKT-dependent increase in mTOR signaling and attenuated muscle atrophy by downregulating MuRF1 and *MAFbx*. However, the present study was limited to measuring the effect of LE treatment on attenuating muscle atrophy by decreasing MuRF1 and *MAFbx* expression. Therefore, the investigation of the affect the LE treatment on skeletal muscle protein ubiquitination might be interesting as a future study.

In this study, the LE-supplemented group demonstrated enhanced mtDNA levels and an upregulation of genes related to mitochondrial biogenesis and OXPHOS. It is well known that AMPK acts as a crucial regulator of biological functions in skeletal muscle, such as in muscle atrophy, lipid metabolism, myokine secretion, and mitochondrial function. PGC-1α, as a downstream transcription regulator mediated by AMPK, is associated with abundant biological pathways in skeletal muscle [48]. In this study, LE supplementation was shown to activate AMPK, thereby enhancing mitochondrial function and biogenesis (Fig. 5). Mitochondrial dysfunctions, such as the loss of mitochondria, changes of mitochondrial morphology, increased ROS production, and impaired mitochondrial oxidative phosphorylation are associated with muscle atrophy [14]. Following the deposition of intramyocellular lipids, a reduction in mitochondrial number and elevation of ROS production may occur. This results in impaired muscle function and reduced oxidative capacity of the muscles [49]. We found that the protective activity of LE against sarcopenic obesity was accompanied by an increase in mitochondrial content and function. LE also increased PGC-1α, which mediates mitochondrial biogenesis and inhibits muscle atrophy by suppressing FoxO3.

In conclusion, LE can mitigate obesity-induced skeletal muscle atrophy in C2C12 myotubes and HFD-induced obese mice. Supplementation with LE effectively increased muscle mass, strength, and function in obese mice. LE also decreased lipid accumulation in the skeletal muscles and inhibited inflammatory cytokines. The mechanism responsible for the protective effect of LE against muscle atrophy is related to an increase in mTOR signaling for protein synthesis and the activation of PGC-1α for the improvement of mitochondrial function. Shikonin, the main component of LE, partly mediated the anti-muscle wasting effect of LE. Therefore, LE is a potential candidate for the prevention or treatment of obesity and obesity-associated sarcopenia.

## Supplemental Materials

Supplementary data for this paper are available on-line only at http://jmb.or.kr.
