# Supplementary material for: Gromwell (Lithospermum erythrorhizon) Attenuates High-Fat-Induced Skeletal Muscle Wasting by Increasing Protein Synthesis and Mitochondrial Biogenesis
Source: J Microbiol Biotechnol. 2023 Dec 30;34(3):495–505. doi: 10.4014/jmb.2311.11034 (PMC11016769; doi:10.4014/jmb.2311.11034)
Supplement: Supplementary file 1 [file jmb-34-3-495-supple.pdf]

J. Microbiol. Biotechnol.
https://doi.org/10.4014/jmb.2311.11034

Supplementary Tables

Gromwell (*Lithospermum erythrorhizon*) attenuates high-fat-induced skeletal muscle wasting by increasing protein synthesis and mitochondrial biogenesis

Ji-Sun Kim [1,2,3], Hyunjung Lee [1], Ahyoung Yoo [1], Hang Yeon Jeong [1], Chang Hwa Jung [1,4], Jiyun Ahn [1,4*], Tae-Youl Ha [1,4*]

[1] Aging and Metabolism Research Group, Korea Food Research Institute, 245 Nongsaengmyeong-ro, Iseo-myeon, Wanju-gun, Jeollabuk-do 55365, Republic of Korea

[2] Department of Biotechnology, College of Life Science and Biotechnology, Korea University, 145 Anam-ro, Seongbuk-gu, Seoul 02841, Republic of Korea

[3] BK21 FOUR Institute of Precision Public Health, Interdisciplinary Program in Precision Public Health, Korea University, Seoul 02841, Republic of Korea

[4] Department of Food Biotechnology, University of Science and Technology, 245 Nongsaengmyeong-ro, Iseo-myeon, Wanju-gun, Jeollabuk-do 55365, Republic of Korea

*

Table S1. Primers and sequences for qPCR.

Gene	Forward (5'-3')	Reverse (5'-3')
MuRF1	AGAAGGCTCAGAAGTTGGAGAC	GGATGGAATACATCTGGTGTTTCG
TNF-α	TGTGGTCCGAGTTGGTATCTT	GCACCTCCAATCACTGTGCC
COX5a	CCGCAAGGGAAAGATGAAAGA	TCGTTTGGTTTCGGGGTTTC
PGC-1α	CACCAAACCCACAGAAAACAG	GGGTCAGAGGAAGAGATAAAGTTG
NRF1	AATGTCCGCAGTGATGTCC	GCCTGAGTTTGTGTTTGCTG
NRF2	TGAAGTTCGCATTTTGATGGC	CTTTGGTCCTGGCATCTCTAC
TFAM	CACCCAGATGCAAAACTTTCAG	CTGCTCTTTATACTTGCTCACAG
NDUFS8	GTTCATAGGGTCAGAGGTCAAG	TCCATTAAGATGTCCTGTGCG
SDHB	ACCCCTTCTCTGTCTACCG	AATGCTCGCTTCTCCTTGTAG
UQCRC1	ATCAAGGCACTGTCCAAGG	TCATTTTCCTGCATCTCCCG
COX5β	ACCCTAATCTAGTCCCGTCC	CAGCCAAAACCAGATGACAG
ATP5a1	CATTGGTGATGGTATTGCGC	TCCCAAACACGACAACTCC
18S	GCCAGCCTCTCCTGATGT	GGGAACACAAAAGACCTCTTCTGG

MuRF1, muscle RING-finger protein-1; TNFα, tumor necrosis factor alpha; Cox5a, cytochrome c oxidase 5a; *PGC-1α*, peroxisome proliferator-activated receptor gamma coactivator 1-alpha; *NRF1* nuclear respiratory factor 1; *NRF2*, nuclear factor erythroid-derived 2-related factor 2; *TFAM*, mitochondrial transcription factor A; *NDUFS8*, NADH dehydrogenase [ubiquinone] iron-sulfur protein 8; *SDHB*, succinate dehydrogenase [ubiquinone] iron-sulfur subunit; *UQCRC1*, ubiquinol-cytochrome c reductase core protein 1; *COX5β*, cytochrome c oxidase subunit 5β; *ATP5a1*, ATP synthase subunit alpha; *18S*, 18S ribosomal

Table S2. Compositions of the experimental diets.

.(g/kg diet)

Experimental diet (g)	ND[1)]	HFD	HFD+LE
Casein	200	200	200
Corn oil	50	50	50
Lard	-	200	200
Cholesterol	-	5	5
Corn starch	350	145	138.24
Sucrose	300	300	300
Cellulose	50	50	50
Mineral mixture[2)]	35	35	35
Vitamin mixture[3)]	10	10	10
Methionine	3	3	3
Choline bitartrate	2	2	2
LE[4)]	-	-	6.76

[1)] ND, normal diet; HFD, high-fat diet; HFD+LE, *Lithospermum erythrorhizon*-supplemented HFD

[2)] AIN-76 mineral mixture contained (in g/kg of mixture): dibasic calcium phosphate, 500.0; sodium chloride, 74.0; potassium citrate monohydrate, 220.0; potassium sulphate, 52.0; magnesium oxide, 24.0; manganous carbonate, 3.5; ferric citrate, 6.0; zinc carbonate, 1.6; cupric carbonate, 0.3; potassium iodate, 0.01; sodium selenite, 0.01; chromium potassium sulphate, 0.55; finely powdered sucrose, 118.03.

[3)] AIN-76A vitamin mixture contained (in g/kg of mixture): thiamine HCl, 0.6; riboflavin, 0.6; pyridoxine HCl, 0.7; niacin, 3.0; d-calcium pantothenate, 1.6; folic acid, 0.2; d-biotin, 0.02; cyanocobalamin (vitamin B_{12}), 1.0; dry vitamin A palmitate (500,000 U/g), 0.8; dry vitamin E acetate (500 U/g), 10.0; vitamin D_3 trituration (400,000 U/g), 0.25; menadione sodium bisulphite complex, 0.15; finely powdered sucrose, 981.08.

[4)] *Lithospermum erythrorhizon* ethanol extract, solid contents 0.37 ± 0.05 g/mL.
